# Idiopathic Pulmonary Comorbidities and Mechanisms

**DOI:** 10.1155/2021/3963659

**Published:** 2021-10-13

**Authors:** Maricica Pacurari, Amal Mitra, Timothy Turner

**Affiliations:** ^1^Department of Biology, College of Science, Engineering, and Technology, Jackson State University, Jackson, MS 39217, USA; ^2^Department of Epidemiology and Biostatistics, School of Public Health, Jackson State University, Jackson, MS 39217, USA

## Abstract

Idiopathic pulmonary fibrosis (IPF) is a disease with an unknown etiology mainly characterized by a progressive decline of lung function due to the scarring of the tissue deep in the lungs. The overall survival after diagnosis remains low between 3 and 5 years. IPF is a heterogeneous disease and much progress has been made in the past decade in understanding the disease mechanisms that contributed to the development of two new drugs, pirfenidone and nintedanib, which improved the therapeutic management of the disease. The understanding of the cofactors and comorbidities of IPF also contributed to improved management of the disease outcome. In the present review, we evaluate scientific evidence which indicates IPF as a risk factor for other diseases based on the complexity of molecular and cellular mechanisms involved in the disease development and of comorbidities. We conclude from the existing literature that while much progress has been made in understating the mechanisms involved in IPF development, further studies are still necessary to fully understand IPF pathogenesis which will contribute to the identification of novel therapeutic targets for IPF management as well as other diseases for which IPF is a major risk factor.

## 1. Introduction

Pulmonary fibrosis (PF) remains a devastating lung disease with a prognosis of 3 to 5 years of survival after diagnosis. The high morbidity and mortality rate is mostly due to limited successful treatment options except for lung transplants [1]. Worldwide, the incidence, prevalence, and mortality rate of PF appeared to increase over time [2,3]. A systematic review study by Hutchinson et al. [2] on IPF incidence and mortality data from 21 countries between 1968 and 2012 determined that both the incidence and mortality rates beyond the year 2000 increased worldwide. In the USA alone, from 1979 to 2003, the age-adjusted mortality rate increased as well [4,5]. However, a more recent study by Jeganathan et al. [6] found that between 2004 and 2017, the age-adjusted mortality rate for IPF decreased. The exact cause of the mortality rate decrease is not known yet; nevertheless, a reduction in smoking is thought to have a role in the observed decline of the mortality rate of IPF in the study by Jeganathan [6]. The causes of PF are not always known (idiopathic, IPF); however smoking; genetics; and environmental and occupational pollutants including cement, silica, dust, asbestos, and certain types of nanoparticles (NP) such as carbon and metal oxides all contribute to the development of PF and other interstitial lung diseases (ILD) [7].

## 2. Pulmonary Fibrosis Comorbidities

Lung fibrosis is a high risk factor for several other lung diseases. Approximately, 30–40% of patients with IPF die due to other comorbidities. Most common morbidities are lung cancer (LC), bacterial pneumonia, arterial pulmonary hypertension (PAH), gastroesophageal reflux (GER), diabetes mellitus (DM), obstructive sleep apnea (OSA), and viral infection. IPF is a progressive and complex disease as it is a risk factor as well as an add-on risk factor to the above indicated comorbidities [7,8].

### 2.1. Lung Cancer

Progressive scarring deep in the lungs happens during the development of IPF and at the same time, the scarring is also a risk factor for LC. Patients with IPF develop LC and patients with LC develop IPF [8,9]. LC prevalence in patients with IPF shows variability such as it can increase from 3.7% up to 20% with an overall cumulative incidence that can range from 3.3% to 15.4% and 54.7% as the time after IPF diagnosis increases from 1 to 5 to 10 years [10,11].

Conversely, IPF prevalence is about 1.3% in patients with LC [12]. However, when IPF is combined with emphysema (CPEE), the prevalence of CPEE in patients with LC is much higher than in patients with IPF alone [12]. The prevalence of LC in IPF patients appears to depend on ethnicity as well. For example, in Denmark, the analysis of a cohort of patients diagnosed with IPF between 2003 and 2009 identified that 6% of patients with IPF also developed LC [13], whereas in a Japanese cohort, a 20.4% incidence of LC was found in patients with IPF [11]. While a direct comparison of LC incidence in IPF patients is difficult to assess mostly due to heterogeneity of population cohorts in the studies thus limiting IPF diagnosis; nevertheless, the existing evidence suggests that LC is a greater risk factor in IPF patients. Moreover, LC shortness overall survival of patients with IPF [13].

### 2.2. Pulmonary Arterial Hypertension

Often patients with IPF develop pulmonary arterial hypertension (PAH) which negatively impacts survival [14]. The median survival for patients with IPF and systolic pulmonary arterial pressure (SPAP) of >50 mmHg could reach 0.7 years versus 4.8 years for those with a SPAP of ≤35 mmHg [15]. While there have been investigations on how to pharmacologically address PAH in IPF patients to improve survival, little progress has been made [15,16]. A clinical study investigating whether sildenafil in combination with pirfenidone given to patients with advanced IPF and PAH may provide a pharmacological benefit to improve survival rate showed no benefit [16].

### 2.3. Diabetes

Diabetes is a comorbidity in IPF and its prevalence in IPF patients appears to depend also on ethnicity [13,17]. Diabetes prevalence in Danish IPF patients in a cohort study between 2003 and 2009 showed that at the beginning of the study there were 9% patients with diabetes and an additional 8% of the patients were diagnosed with diabetes during the follow-up of the study; thus a total of 17% of patients with IPF had also diabetes. The presence of diabetes at the beginning of the study was associated with higher IPF mortality compared to those who did not have diabetes at the beginning of the study [13]. In the US, a retrospective population-based study using CDC database from 2007 to 2017 identified IPF in 0.65% of diabetic patients and 0.80% in non-diabetic patients [17]. Most body organ functions such as those of kidneys, retina, heart, brain, and lungs are profoundly affected by diabetes [18]. Lung function is already reduced in patients with diabetes which is further complexed by risk factors such as race with Caucasians having a lower lung function compared to African-Americans and Hispanics [19]. Type I diabetes is also an independent risk factor for PF. Hu et al. [20] using a mouse model of type I diabetes showed that clinical and histopathological characteristics correlated with type I diabetes and PF. Moreover, induction of type I diabetes led to increased expression of lung fibrosis markers CTGF and fibronectin in diabetic lungs of the animal model [20].

### 2.4. OSA

OSA is now recognized as an important IPF comorbidity [21]. OSA prevalence can be high as much as 88% of IPF patients are also diagnosed with OSA [22]. Moreover, OSA is also associated with higher morbidity and mortality in IPF patients [23]. Patients with IPF often report symptoms such as fatigue and very low sleep quality and sleep-related breathing disorders (SRBD) including hypoventilation [23]. Studies suggest an early OSA diagnosis in IPF patients and its treatments with continuous positive airway pressure (CPAP) results in a significant improvement of sleep quality and also a reduction in mortality [24,25]. The relationship between OSA and IPF is due to common mechanisms such as hypoxia and hypoxemia. During OSA, hypoxia does happen which then contributes to pulmonary constriction and the development of hypoxia-induced pulmonary hypertension (HPH). Also, hypoxia activates an array of intracellular signaling pathways such as those of Ca^2+^-mediated cell proliferation and migration, inflammation, and chemotaxis, thus exacerbating lung tissue which is already limited in its function due to fibrosis [26]. Hypoxemia episodes during intermitted breathing of OSA lead to oxidative stress (OS) and formation of reactive oxygen species (ROS) which induce inflammation and damage to vascular endothelium thus contributing to vascular vasoconstriction alone or in conjunction with sympathetic-mediated signaling and thus further complicating the effects of PF [27]. Therefore, these studies suggest that managing OSA in PF patients may improve PF management.

## 3. Mechanisms of PF

Recent studies showed an increase in the prevalence of IPF in the USA and worldwide [28–30]. This increase in IPF occurrence is mostly attributed to the heterogeneity of the disease and mechanisms of pathogenesis that are not fully understood yet [31,32]. While IPF pathobiology is multifactorial, cellular and molecular mechanisms including cellular transformation, epithelial–mesenchymal transition (EMT), ROS, overactive TGF*β*, NF-*κ*B, and MAPK signaling in the lung fibroblasts and epithelial cells all play important roles in the disease development [33,34]. Some of these mechanisms also overlap the IPF-associated comorbidities such as LC.

### 3.1. Cellular Factors Contributing to LC Development in IPF

The type of LC that develops in IPF patients is mostly nonsmall cell lung cancer (NSCLC). How IPF influences NSCLC development is not exactly known yet. However, there are several common risk factors including genetic, molecular, and cellular that coinfluence each other.

#### 3.1.1. Cellular Transformation

A major characteristic in the development of IPF is an increase in myofibroblast number and activation [35]. The activated myofibroblasts secrete extracellular matrix (ECM) that is rich in collagen type I (Col I) and *α*-smooth muscle actin (*α*-SMA). What contributes to myofibroblast increase and activation is not exactly known. However, processes such as continuous lung injury, fibroblast proliferation, and cell transdifferentiation are factors that contribute to myofibroblast activation. Single-cell sequencing analysis in a lung fibrosis animal model produced a 49-gene signature that defined activated fibroblasts; however, none of the signature genes were specific only to activated fibroblast compared to control fibroblast suggesting that activated fibroblasts are not the only cell type playing a key role in lung fibrosis but an increase in other cells types such as those of macrophages, dendritic cells, or myeloid proliferating cells may play a role in lung fibrosis pathogenesis [36]. Alveolar epithelial cells (AEC) undergo EMT under TFG*β*1 stimulation [37]. TFG*β*1 multifactorial signaling is complex such as on the background of IPF signals both fibrotic and carcinogenesis pathways [38]. ECM regulation is also a complex process. For example, analysis of *Col 5a2*, *Acta2*, and *ltbp2* genes in cells from control and bleomycin-induced mouse lung fibrosis showed their expression in both activated and nonactivated fibroblast, as well as other cells populations. Moreover, to complicate lung biology, ECM regulation, myofibroblast activation, and EMT are processes that are common to both IPF and LC. In fact, nintedanib, a second-line treatment for LC is also used for the treatment of IPF [39,40].

#### 3.1.2. Alveolar Epithelial Cells (AEC)

PF is a multifactorial disease. Hallmarks of PF are the formation of fibrotic lesions containing collagen and ECM overproduction. While lung fibroblasts are the main players in PF, AEC also contribute to lung interstitial space remodeling [41]. Animal studies indicate that injury to alveolar epithelium plays a key role in the initial phase of the fibrotic process while GWAS studies indicate that many other cellular and molecular factors such as macrophages, dendritic cells, or mTOR and mitotic spindle-assembly genes KIF15 and MAD1L1 play a role in IPF susceptibility [41–44]. AEC undergo EMT becoming myofibroblast under continuous injury or presence of TGF*β* and thus contribute to fibrotic signaling [41,45]. The newly proposed mechanism in IPF development is that AEC contribute to fibrotic signaling either via a coexisting injury to AEC alone or along with ineffective repair mechanisms that contribute to the formation of nonfunctional alveolar units that gradually are replaced with fibrotic tissue that is nonfunctional. Injury to alveolar epithelium, capillary endothelium, or ECM, as well as alveolar macrophages and neutrophils, elicit inflammatory signals that contribute to lung parenchyma structural architecture modifications. These modifications also include the accumulation of mesenchymal cells and fibroblasts within alveolar space and the formation of an inflammatory milieu that potentiates fibrotic responses [46]. The pathogenesis of IPF is complex such as involving both cellular and molecular factors; however, the role of AEC in IPF emerges more prominently than presumably thought [46].

#### 3.1.3. Macrophages

The population of macrophages in the lungs can exist either as (1) alveolar macrophages (AM) that reside in the alveoli or (2) interstitial macrophages (IM) that reside in the lung parenchyma [47,48]. Both of these two groups of macrophages have distinct functions: AM are responsible for immune functions by producing both pro- and anti-inflammatory cytokines, whereas IM maintain immune balance within the lungs [49]. Pulmonary macrophages play a role in the pathogenesis of IPF particularly given their extraordinary plasticity. Macrophages can polarize into activated macrophages (M1) that mainly regulate an initial inflammatory response and also M1 can polarize into alternatively activated (M2) macrophages that mediate tissue repair and remodeling particularly during the resolution phase of inflammation [50]. Proinflammatory cytokines (IFN*γ*, TNF*α*, IL-1*β*, IL-6, Il-8, IL-12, IL-13, MIP-1*α*), LPS, signaling pathways (AKT2, NOTCH1/2), transcription factors (AP-1, NF-*κ*B, STAT1), and miRNAs (miR-155, let-7c, miR-124, miR-223) all regulate M1 activation. Once activated, M1 macrophages can further produce inflammatory cytokines such as those of the CXCL group (CXCL1-3, CXCL-5, or CXCL8-10) and contribute to the formation of ROS or reactive nitrogen species (RNS) that further damage the tissue [51,52]. M2 macrophages are activated by anti-inflammatory cytokines (IL-4, IL-10, IL-13), growth factors (TGF*β*, CTGF, PDGF), or transcription factors (PPAR*γ*); thus, any stimuli that induce the production of these factors play a key role in M2 activation [53,54]. Moreover, depending on the type of the signals, M1/M2 can switch forth and back and thus greatly modulate tissue inflammation and injury responses [55]. In acute lung injury (ALI) or acute respiratory distress syndrome (ARSD), M1 are actively polarized and further propagate inflammation by producing even more inflammatory signals and thus contributing to severe lung inflammation while M2 macrophages are decreasing [56,57]. M2 macrophages mediate lung tissue injury repair and remodeling by producing anti-inflammatory cytokines and also phagocytizing cellular debris. These M2-mediated processes play a key role in controlling and limiting tissue inflammation [58,59]. Acute inflammation in the initial phase of Ali/ARDS further potentiates lung injury through mediating the release of more cytotoxic mediators that facilitate leukocytes or macrophages infiltration and activation of pulmonary epithelial or endothelial cells which through complex signaling pathways contribute to the development of fibrogenic responses in the lungs [60–62]. Thus, macrophages through their signaling pathway modulate the lung environment and greatly and influence disease outcome.

#### 3.1.4. Pulmonary Fibroblasts

A key feature of PF is the deposition of fibrous tissue within the lung space which subsequently leads to a smaller functional lung that presents an abnormal architecture characterized by collapsed airspaces. In fibrotic lungs, fibroblasts are activated and usually form small clusters that actively synthesize collagen and other ECM molecules in small patches called fibrotic foci that are widely spread within the fibrotic lung parenchyma [63]. Studies have shown that there is a difference between fibroblasts from normal lungs versus fibrotic lungs. In some studies, fibroblasts from fibrotic lungs proliferate faster than fibroblasts from normal lungs [64,65]. Other studies found that fibroblasts from fibrotic lungs have a lower growth rate and increased apoptosis, and other studies found that fibrotic fibroblasts have an increased migration into ECM that is regulated by CD44 and hyaluronan synthase 2 (HAS2) [66,67]. While there is a difference regarding fibroblasts proliferation capacity in fibrotic lungs versus normal lungs, there is a consensus that fibroblasts from fibrotic lungs undertake an active profibrotic role by synthesizing fibrotic factors mainly Col I as well as other ECM components including TGF*β*1, matrix metalloproteinase-9 (MMP-9), and tissue inhibitors metalloproteinases (TIMPs) [66,67]. What factors contribute to fibroblasts activation has been the focus of intense research for the past decade and those studies contributed to an understanding of what contributes to fibroblasts profibrotic phenotype switch. Those studies found that HAS2, CD44, TGF*β*, TNF*α*, lumican, IL-17, TLR4, or S100A4 plays a key role in fibroblast activation and migration [68–73].

### 3.2. ECM

Although ECM is a noncellular integral component of all tissues and organs by providing a physical framework that is very important for all cellular, biochemical, and mechanical signaling for tissue development as well as for homeostasis, ECM is a dynamic structure composed of proteoglycans (PG) and fibrous proteins (collagen, elastin, fibronectin, and laminin) that continuously undergo regulated remodeling as an integral process of normal tissue functioning. In the lungs, ECM is part of the basement membrane and interstitial space where fibroblasts reside and produce ECM components. The origin of fibroblast remains debatable as some studies suggest that lung epithelial cells undergo a mesenchymal transformation in the injured lungs, while others found that alveolar epithelial cells and bone marrow progenitor cells contribute to fibroblast population in fibrotic lungs but neither one of them is a major source of fibroblasts, while others found that stromal cell populations with characteristics of pericytes are a source of myofibroblasts in a bleomycin mouse model of fibrotic lungs [74–76]. Dysregulated ECM remodeling plays a key role in IPF [77]. Fibrotic lungs show heterogeneity and are usually characterized histologically with normal and fibrotic tissue, epithelial/alveolar damage, and fibrotic foci. At an advanced stage of IPF, the lung alveoli are nearly wiped out which clinically entails lung transplant [78]. Abnormal tissue remodeling is a consequence of unbalanced signaling following an injury within the lungs toward mediators that are inflammatory (IL-6 and TNF*α*) and profibrotic produced by activated macrophages and fibroblasts [79]. Indeed, IPF is a variable disease since genetic predisposition, age, and other risk factors do not always culminate with the disease, yet lung function decline indicates that acute lung inflammation precedes fibrotic process thus suggesting that the development of IPF is a complex process within the lungs where fibroblasts, epithelial, and immune cells interact and influence the pathology within lungs [80,81]. Studies indicate the presence of a structural, mechanical, and biochemical difference between fibrotic and normal healthy ECM thus fully understanding how cellular and molecular cues together with lung environment modulate ECM toward pathological signaling may represent a novel therapeutic approach for the management of IPF [82–84].

### 3.3. Molecular Mechanisms

#### 3.3.1. ROS

ROS through the activation of signaling pathways upstream and downstream of the profibrotic growth factor TGF*β* play a key role in the pathogenesis of PF [85,86]. MAPK ERK/1/2 and p38 are redox-sensitive kinases and known to induce translocation of transcription factor NF-*κ*B and modulate the expression of genes including TGF*β* [87,88]. Under pathological conditions, cross talk between NF-*κ*B and TGF*β* signaling exists, for example, TGF*β* promoter contains p65 binding sites and TGF*β* induces p65 phosphorylation [89]. TGF*β* also plays a role in mitochondrial ROS generation which further contributes to the augmentation of TGF*β*-mediated responses independent of the canonical Smad complex. Mitochondrial ROS mediate TGF*β* signaling via activation of MAPK or JNK pathways [90]. Canonical TGF*β*-Smad complex is formed upon TGF*β* binding to its receptor on the plasma membrane and initiating a signaling cascade that leads to interactions of Smad 2/3 and Smad 4 and formation of Smad complex which then translocates into the nucleus and through interactions with other transcription coactivators and corepressors facilitate Smad complex to recognize binding sequences on target genes including profibrotic genes [91]. Although antioxidant therapies have been used in the therapeutic management of IPF, treatment outcomes are less than expected thus suggesting a complex mechanism of ROS signaling in IPF [92].

#### 3.3.2. NOX4

ROS producing NADPH oxidase isoform 4 (NOX4) in complex with p22phox plays a key role in the generation of ROS and activation of redox-sensitive signaling pathways in both normal and pathophysiological conditions [93]. In human fibrotic lungs, NOX4 is highly expressed [94]. Mechanistic studies showed that NOX4-induced ROS modulate pulmonary myofibroblast activation in the presence of TGF*β* and PDGF [95]. In the mouse model of bleomycin-induced lung fibrosis, local inhibition of NOX4 either with siRNA or nonselective pan-NOX inhibitor diphenyleneiodonium (DPI) or small molecule inhibitor GKT137831 resulted in limited collagen deposition within lungs and diminished the extent of PF [95,96]. NOX4 signaling is complex. NOX4 activity is increased by TGF*β* and since TGF*β* is overexpressed in fibrotic lungs, NOX4-dependent ROS production is regulated by TGF*β* and further promotes fibrogenic responses [95,97]. Studies indicate that ROS produced via NOX4 play a key role in the pathogenesis of PF and that the current NOX4 inhibitor, GKT137831, is tested in Phase 2 clinical trial [98].

#### 3.3.3. TSP-1

TSP-1 is an adhesive homotrimeric glycoprotein of the ECM that interacts with cell receptors, growth factors, and cytokines. TSP-1 is also a major activator of latent TGF*β* [99,100]. TGF*β* in complex with TSP-1 is biologically active and favors TGF*β* binding to cell receptors [101]. Platelets, endothelial cells, smooth muscle cells, neutrophils, monocytes, macrophages, alveolar macrophages, fibroblasts, and epithelial cells all produce TSP-1 [102–106]. TSP-1 regulates multiples cellular processes such as cell migration, cell adhesion, cell proliferation, apoptosis, coagulation, wound repair, and fibrosis [107,108]. TSP-1 signaling is complex. Genetic or pharmacological manipulation of TSP-1 alone or along with *β*6 integrin revealed severe inflammation and epithelial hyperplasia particularly in the lungs suggesting a key role of TSP-1 in normal lung physiology [109,110]. Pathological conditions such as injury, diabetes, stress, or inflammation stimulate the production of TSP-1 which plays a key role in the activation of macrophages in the wound healing process and also activation of latent TGF*β* which favors fibrotic responses and organ dysfunction [111–113]. High glucose potentiates TSP-1 activation of TGF*β* [114]. In animal models of diabetes, TSP-1 plays a key role in TGF*β*-mediated organ injury [115,116]. In human fibrotic lung biopsies, TSP-1 is highly expressed in fibrotic areas and colocalizes with SPA in type II pneumocytes. Alveolar macrophages and regenerative epithelium of fibrotic lesions also express high levels of TSP-1 [117,118]. Proteomic analysis of plasma from patients with IPF identified TSP-1 among 9 proteins to be highly expressed compared to controls [119]. In an animal model of pulmonary fibrosis, alveolar macrophages are detected in the fibrotic foci and express high levels of TSP-1 and TGF*β*. Blocking TSP-1 interaction with its receptor, CD36, reduces pulmonary inflammation and fibrosis [120]. TSP-1 also interacts with CD47, LRP, CRT, PPAR*γ*, and *α*4*β*1 cell surface proteins and facilities focal adhesion disassembly, chemotaxis, and adhesion of T-cells [120–123]. TSP-1 is an immediate response gene of TGF*β* and PDGF, and its promoter also contains binding sites for NF-*κ*B and AP-1. Both of these transcription factors are also activated by ROS thus suggesting a cross talk between signaling pathways that regulate both ROS and TSP-1-mediated responses [124–126]. Mechanical forces upregulate TSP-1 in developing sheep lungs or platelets [127,128]. Hypoxia increases TSP-1 in fibroblasts of patients with systemic scleroderma, pulmonary artery smooth cells, endothelial cells, and pulmonary fibroblast [129]. However, it remains unclear how TSP-1 is mechanistically regulated, and limited studies show that transcription factor HIF-2*α* is a direct activator of TSP-1 whereas c-Myc downregulates TSP-1 [130,131]. PI3K/AKT, ERK1/2, and p38 MAPK regulate TSP-1 in various cell types [132–134]. These studies indicate that TSP-1 interacts with many factors and thus plays a key role in cell adhesion, cell migration, angiogenesis, and tissue remodeling and fibrosis [134]. In animal model of bleomycin-induced lung fibrosis, TSP-1 is highly expressed and blocking TSP-1 with a synthetic peptide reduces pulmonary fibrosis progression [135]. Although numerous studies indicate that TSP-1 has a role in the pathogenesis of fibrotic disease and other cellular process, no specific TSP-1 inhibitor for lung fibrosis has been developed. However, TSP-1 is a major regulator of angiogenesis and a TSP-1 mimetic angiogenesis inhibitor, ABT-510, is tested in preclinical trials [136].

#### 3.3.4. Notch

The Notch family is a member of the transmembrane receptors signaling and is essential for lung development and homeostasis. Notch proteins are also major regulators of progenitor cell niche for lung repair mainly after injury [137]. The Notch family is represented by four Notch receptors numbered 1 to 4 (1–4) and five Notch ligands: Delta-like 1, 3, 4 and Jagged 1 and 2 [138]. Dysregulated Notch signaling has been implicated in tissue fibrosis [139–141]. For example, in bleomycin-induced mouse lung fibrosis, Notch 1 and Jagged 1 are highly expressed in fibrotic lungs compared to control normal lungs, and mesenchymal Notch 1 conditional genetic deletion partially diminished bleomycin-induced lung fibrosis and reduced myofibroblast differentiation. However, no difference in immune and inflammatory cells was observed in the fibrotic and control mouse lungs [142]. Another study showed that Notch 1 inhibition with DAPT attenuated lung fibrosis in the bleomycin mouse model, decreased *α*-SMA and Col I, and downregulated PDGFR*β* and ROCK1 expression [143]. However, inhibiting PDGFRs in an irradiated mouse model of lung fibrosis also attenuated lung fibrosis progression [144]. The results of these studies indicate that although Notch 1 was absent in mesenchymal cells, other cells and signaling pathways may compensate for Notch 1. Some signaling pathways that compensate for Notch are those of TSP-2, Notch 3 and Jagged 1 and canonical Shh signaling which have been shown to mediate cross talk between epithelial and myofibroblast differentiation [145,146]. Although these studies indicate Notch as a promising target, future sophisticated research will contribute to delineating the exact role of the Notch family in lung fibrosis.

#### 3.3.5. microRNAs

Numerous studies indicate that microRNAs (miRs) are involved in the development of IPF. Analysis of lung biopsies revealed differential expression of microRNAs. Studies showed that in rapid-progressive IPF lungs, 11 miRs were increased and 36 miRs were decreased compared to control normal lungs. miR-302c, miR423-5p, miR-210, miR-376c, and miR-185 were found to be highly expressed in rapid-progressive IPF compared to slow-progressing IPF whereas miR processing components, Ago1 and Ago2, levels were lower in the rapid-progressive IPF compared to slow progressive IPF or control normal lungs [147,148]. Moreover, analysis of serum from patients with IPF showed increased miR-21 and miR-155 and decreased miR-101-3p. The levels of these microRNAs correlated with forced vital lung capacity (FVC) and radiological analysis [149]. Human fibrotic lungs analysis showed an increased level miR-21 and mechanistic studies showed that miR-21 plays a key role in EMT and fibroblast activation [149,150]. Human fibrotic lungs and silica-induced mouse fibrotic lungs showed low levels of microRNA let-7d and its level correlated with lung function [151]. microRNAs regulation is complicated. For example, TGF*β* decreases microRNA let-7d, whereas exogenous let-7d directly inhibits TGFBR1 and its target genes [152]. Moreover, *in vivo* inhibition of let-7d resulted in alveolar septal thickening and increased Col1A and HMGA2 expression whereas i*n vitro* inhibition increased the level of HMGA2 [153]. TGF*β* is a key driver of pathogenesis in pulmonary fibrosis as it stimulates the production of ECM whereas let-7d has opposing effects on ECM [154]. In pulmonary fibroblasts, TGF*β* suppresses miR-29 whereas exogenous miR-29 decreases TGF*β*-induced Col1A1 [155]. Exogenous administration of miR-542-5p or miR-489 attenuates fibrotic lesions formation in the lungs of a silica mouse model of lung fibrosis [156,157]. Mechanistically, miR-542-5p has been shown to bind 3′-UTR of integrin *α*6 and reduce FAK/PI3K/AKT signaling and fibroblasts activation [156]. miR-1343 has been shown to bind 3′-UTR of TGF*β*R1 and TGF*β*R2 [158].

While numerous mechanistic studies show how microRNAs play roles in PF their breakthrough in PF diagnostic and therapeutic is still at an early stage of such promising clinical approaches. Nevertheless, microRNAs potential in diagnostic is already available for clinical use for some diseases such as thyroid and pancreatic cancer, cardiovascular disease, and osteoporosis [159].

## 4. Conclusion

PF is a multifactorial disease with unknown causes that remains with a low survival (3–5 years) after diagnosis and represents a risk factor for other lung diseases particularly LC. Numerous studies have provided an understanding of PF mechanisms and have contributed to the development of two most recent antifibrotic drugs, pirfenidone and nintedanib, that offer a therapeutic efficacy in the management of PF. However, these two drugs do not fully stop the progression of the disease, suggesting that additional studies are necessary to fully understand PF pathogenesis and as presented in the present review cellular and molecular mechanisms including AEC and microRNAs may contribute to the development of additional therapeutic targets for PF management.

## Data Availability

No data were used in the study.
